# RETRACTED: Khalil et al. Orientin, a Bio-Flavonoid from *Trigonella hamosa* L., Regulates COX-2/PGE-2 in A549 Cell Lines via miR-26b and miR-146a. *Pharmaceuticals* 2022, *15*, 154

**DOI:** 10.3390/ph19071054

**Published:** 2026-07-08

**Authors:** Hany Ezzat Khalil, Hairul-Islam Mohamed Ibrahim, Emad A. Ahmed, Promise Madu Emeka, Ibrahim A. Alhaider

**Affiliations:** 1 Department of Pharmaceutical Sciences, College of Clinical Pharmacy, King Faisal University, Al-Ahsa 31982, Saudi Arabia; pemeka@kfu.edu.sa (P.M.E.); ialhaider@sfda.gov.sa (I.A.A.); 2 Department of Pharmacognosy, Faculty of Pharmacy, Minia University, Minia 61519, Egypt; 3 Department of Biological Sciences, College of Science, King Faisal University, Al-Ahsa 31982, Saudi Arabia; himohamed@kfu.edu.sa (H.-I.M.I.); eaahmed@kfu.edu.sa (E.A.A.); 4 Department of System Biology, Pondicherry Center for Biological Science and Educational Trust, Kottakuppam 605104, India; 5 Lab of Molecular Physiology, Department of Zoology, Faculty of Science, Assiut University, Assiut 71515, Egypt; 6 Research and Development, Saudi Food and Drug Authority, Riyadh 13312, Saudi Arabia

This journal retracts the article titled “Orientin, a Bio-Flavonoid from *Trigonella hamosa* L., Regulates COX-2/PGE-2 in A549 Cell Lines via miR-26b and miR-146a” [1], cited above.

Following publication, concerns were brought to the attention of the Editorial Office regarding the integrity of a number of images presented in this publication [1].

Adhering to our standard procedure, an investigation was conducted by the Editorial Office and Editorial Board that identified indications of inappropriate editing and duplication within Figure 6, published in [1]. While the authors collaborated during this process, they were unable to provide complete, original supporting material for the Editorial Board’s evaluation. Consequently, the Editorial Board has lost confidence in the reliability of the findings and has decided to retract this publication, as per MDPI’s retraction policy (https://www.mdpi.com/ethics#_bookmark30, accessed on 1 May 2026).

This retraction was approved by the Editor-in-Chief of the journal *Pharmaceuticals*.

The second author (H-I M. Ibrahim) informed the journal that he was responsible for the data and image curation. The authors have been informed of this retraction and have indicated their disagreement with this decision.
